# Impact of the Transboundary Interference Inhibitor on RNAi and the Baculovirus Expression System in Insect Cells

**DOI:** 10.3390/insects15060375

**Published:** 2024-05-21

**Authors:** Hao Zheng, Hengfeng Zhao, Haifan Xiong, Mian Muhammad Awais, Songrong Zeng, Jingchen Sun

**Affiliations:** 1 Guangdong Provincial Key Laboratory of Agro-Animal Genomics and Molecular Breeding & Subtropical Sericulture and Mulberry Resources Protection and Safety Engineering Research Center, College of Animal Science, South China Agricultural University, Guangzhou 510642, China; zhenghao_scau@163.com (H.Z.); 19878869550@163.com (H.Z.); constancehf@163.com (H.X.); awaismian31@yahoo.com (M.M.A.); 2 Guangdong Provincial Key Laboratory of Utilization and Conservation of Food and Medicinal Resources in Northern Region, Shaoguan University, Shaoguan 512005, China; zsrdata@163.com

**Keywords:** viral suppressor of RNAi, insect cells, gene regulation, apoptosis

## Abstract

**Simple Summary:**

RNA interference and RNA interference inhibition are natural mechanisms of interaction between viruses and host cells, widely observed in plant and mammal viruses. However, the interaction between these inhibitors and insect cells remains poorly understood. In this study, we employed recombinant baculoviral vectors to introduce RNA interference inhibitors with diverse modes of action into insect Sf9 cells, systematically evaluating their impact on cell cycle progression and gene expression. Furthermore, our findings demonstrate that these suppressor genes can significantly enhance the potential application of insect cell bioreactors for producing recombinant proteins and baculoviruses. Overall, this study elucidates the influence of heterologous RNA interference inhibitors on the mechanism of RNA interference in insect cells while providing novel insights for engineering insect cell bioreactors.

**Abstract:**

RNA interference inhibitors were initially discovered in plant viruses, representing a unique mechanism employed by these viruses to counteract host RNA interference. This mechanism has found extensive applications in plant disease resistance breeding and other fields; however, the impact of such interference inhibitors on insect cell RNA interference remains largely unknown. In this study, we screened three distinct interference inhibitors from plant and mammal viruses that act through different mechanisms and systematically investigated their effects on the insect cell cycle and baculovirus infection period at various time intervals. Our findings demonstrated that the viral suppressors of RNA silencing (VSRs) derived from plant and mammal viruses significantly attenuated the RNA interference effect in insect cells, as evidenced by reduced apoptosis rates, altered gene regulation patterns in cells, enhanced expression of exogenous proteins, and improved production efficiency of recombinant virus progeny. Further investigations revealed that the early expression of VSRs yielded superior results compared with late expression during RNA interference processes. Additionally, our results indicated that dsRNA-binding inhibition exhibited more pronounced effects than other modes of action employed by these interference inhibitors. The outcomes presented herein provide novel insights into enhancing defense mechanisms within insect cells using plant and mammal single-stranded RNA virus-derived interference inhibitors and have potential implications for expanding the scope of transformation within insect cell expression systems.

## 1. Introduction

RNA interference (RNAi), also known as RNA silencing, is a biological regulatory mechanism that precisely silences or downregulates the expression of specific nucleic acids through small RNA molecules [1,2]. This mechanism is widely present in organisms and represents a highly conserved molecular regulatory mode following gene transcription [3]. The fundamental principle of RNAi involves the utilization of exogenous long double-stranded RNAs (dsRNA) or endogenous hairpin-shaped microRNAs, which are processed by the Dicer enzyme (an RNase III enzyme) within cells to generate 19–23 bp small interfering RNAs (siRNAs) or 19–23 nt single-stranded microRNAs (miRNAs) [4]. These spliced siRNAs and miRNAs subsequently bind to various enzymes within cells, forming RNA-induced silencing complexes (RISC) [2,5]. Guided by these small molecules, the RISC can specifically degrade target mRNAs and block their translation, thereby reducing or silencing the expression of target genes [6]. The phenomenon of RNAi was initially discovered in nematodes where researchers effectively silenced endogenous genes homologous to injected dsRNA in *Caenorhabditis elegans* [7]. Subsequent studies have confirmed that RNAi is widespread among fungi, plants, fish, insects, and mammals [8,9,10,11,12]. In cell development processes, RNAi plays a crucial role in gene regulation and serves as an important immune defense mechanism for organisms against exogenous viruses and gene invasion [13,14,15]. The precise regulation of this mechanism is vital for normal growth, development, and reproduction, as well as adaptation to environmental changes and disease resistance [16].

During the extensive evolutionary process between viruses and host organisms, certain viruses have gradually developed a gene structure known as the Viral Suppressor of RNA interference (VSR) to enhance their survival and reproductive capabilities within host cells [17]. These VSRs significantly inhibit RNAi, an antiviral defense mechanism in organisms involving siRNA production, RISC formation, Dicer enzyme cleavage, and other biochemical reactions [18]. Plant and animal viruses have been found to possess various VSRs that often serve as key pathogenic factors for these viruses [19]. VSRs can target multiple steps in the RNAi pathway to impede the transmission and amplification of RNAi, thereby facilitating virus persistence and replication within hosts [20,21]. Further investigations revealed that single-stranded RNA viruses exhibit robust VSR phenomena. However, these related silencers do not display conservation regarding gene sequence or structure, suggesting diverse modes of action and target sites for these VSRs [22,23]. Based on their mode of action, we can broadly categorize VSRs into the following types: (1) binding to dsRNA or siRNA molecules to promote degradation, hindering assembly of the RNA-induced silencing complex (RISC), such as tomato dwarf virus P19 protein and influenza virus NS1 protein [24,25,26]; (2) binding to AGO proteins to inhibit their function, impairing integration of siRNAs into RISC while promoting RISC degradation, such as potato leafroll virus P0 protein and cucumber mosaic virus 2B protein [27,28]; and (3) interacting with Dicer enzymes either through inactivation or degradation processes that hinder siRNA production, examples include *Hepatitis C* virus CORE protein and human immunodeficiency virus 1 transcriptional protein TAT [29,30]. As a survival strategy for viruses within hosts’ cellular environments, VSR enables enhanced viral persistence and reproduction.

As a crucial pathogenic factor of viruses, VSRs possess the ability to resist RNA interference. They may also significantly affect the control of viral infection, the regulation of the cell apoptosis cycle, and the modulation of gene expression. Therefore, investigating the mechanism of RNA interference inhibitors holds paramount significance and value [19,31,32]. However, limited research has been conducted on the impact of VSRs on the modulation of apoptotic gene expression and apoptosis rate in insect cells. In this study, we constructed transient expression vectors and MultiBac expression vectors to achieve early or late expression of RNA-silencing inhibitors in insect Sf9 cells using baculovirus promoters p64 and p10. We systematically explored the impact of plant and mammal RNA virus RNA-silencing inhibitors on insect cells as well as their effects on gene regulation, protein expression, proliferation, and replication of progeny viruses during cell-virus interaction. The findings from this study contribute to further elucidating the molecular mechanisms underlying VSR resistance against RNA interference while optimizing the baculovirus expression system.

## 2. Materials and Methods

### 2.1. Bacterial Strains, Plasmids, MultiBacmid, Reagents, and Genes

*Escherichia coli* (*E. coli*) DH10B, DH5α, and BW23474 were utilized to propagate MultiBac, R6k-γ, and general plasmids. The dual-purpose pie1 plasmid was specifically designed for cloning and achieving high-level protein expression through transient transfection of Spodoptera-derived insect cells. These plasmids contain an ampicillin resistance gene and an hr5 enhancer upstream of the pie1 promoter. The pFBDM and pUCDM plasmids, which are modified MultiBacs with antibiotics resistance genes inserted via transposition (mini-Tn7 and Cre-loxP), were constructed as described in our prior studies [33]. Recombinant MultiBacs were generated by utilizing multiple cloning sites (MCS) present in the plasmids (pFBDM and pUCDM) to introduce multiple foreign genes through specific transposition. *E. coli* Sw106 containing MultiBac, pHelper, and pGB_2_Ωinv was constructed as described in previous reports [33]. *E. coli* Sw106 can directly infect Sf9 cells and enter their cytoplasm to release recombinant MultiBac and generate recombinant baculovirus.

The VSRs utilized in this study were all derived from single-stranded sense RNA viruses. The *p0* gene was obtained from the potato leaf-roll virus, the *p19* gene was acquired from the tomato bushy stunt virus, and the *core* gene corresponded to the CORE protein gene of the *Hepatitis C* virus. The VSR genes were amplified by PCR using specific primers (Table 1) and linked to the corresponding position of plasmid pFBDM through restriction enzymes and DNA ligases. The sequences encoding Firefly luciferase and Renilla luciferase originated from the Dual-Luciferase Reporter Assay System and were employed for exogenous protein expression analysis. Sequences encoding enhanced green fluorescent protein (*egfp*, GenBank: MH891622.1) and mCherry fluorescent protein (*mCherry*, GenBank: MN052904.1) were amplified using respective primers available in our previous report (Table 1), followed by insertion into plasmids as markers for protein expression.

### 2.2. Construction of Transient Expression Plasmids and Transfection into Sf9 Cells

The transient expression plasmids pie1-p0, pie1-p19, and pie1-core were utilized for efficient expression of the VSRs in insect Sf9 cells. These plasmids were constructed from the dual-purpose vector piex/Bac-1, which contains an hr5 enhancer and multiple cloning sites, following established protocols. To generate the recombinant plasmid pie1-p0, the N-terminal fusion protein His-tags gene, designated as the *p0* gene, was amplified using primers p0-F and p0-R in Table 1 and subsequently cloned into the *Bam*H I and *Sac* I restriction enzyme sites of plasmid pie1. Similarly, recombinant plasmids pie1-p10 and pie1-core were obtained using identical methods. *E. coli* DH5α was used for replication of these transient expression plasmids, followed by isolation and purification of the resulting plasmid DNA utilizing a Miniprep kit. The Sf9 cells were cultured overnight, and the cell suspension was prepared using Sf-900™ II SFM medium (Lot.NO: 10902096, Gibco™, Thermo Fisher, Waltham, MA, USA). Monolayer cultures comprising 10^5^–10^6^ cells were established in Petri dishes with a bottom area of 25 mm. The plasmids pie1-p0, pie1-p19, pie1-core, and pie1 (as control) that were successfully constructed were combined with Lipofectamine 3000 Reagent (Lot.NO: L3000001, Thermo Fisher, Waltham, MA, USA) and transfected into Sf9 cells as required.

### 2.3. Extraction and Reverse Transcription of RNA and Quantitative PCR

The transfected insect Sf9 cells were centrifuged at a low speed (4 °C, 2000× *g*) to ensure complete separation of the cell supernatant and cell pellet. The pellet was then resuspended in RNase-free PBS (Lot.NO: AM9624, Invitrogen™, Thermo Fisher, Waltham, MA, USA) and subjected to another round of centrifugation under the same conditions to obtain the purified cell pellet. This process was repeated three times. Trizol reagent was added to the purified cell pellet, and total RNA from the precipitated cells was extracted using an RNA extraction kit with vortex oscillation. The extracted RNA was reverse transcribed into cDNA using Oligo dT (Takara) primers. Subsequently, quantitative PCR analysis was performed on the resulting cDNA using SYBR Premix Ex Taq^TM^ (Tli RNaseH Plus, Takara, Kusatsu, Shiga, Japan) and specific primers listed in Table 2. The expression efficiency of the target gene in insect Sf9 cells was determined by comparing it with that of the internal reference gene (α-Tubulin).

### 2.4. Construction of Recombinant Plasmids for Early and Late Stable Expression of VSRs

To further investigate the impact of ssRNA virus VSRs on apoptosis in insect cells, this study constructed expression cassettes containing multiple target genes (p64-p0, p64-p19, p64-core, p10-p0, p10-p19, and p10-core) using the early promoter of p64 and late promoter of p10 from baculovirus. The target genes were amplified with corresponding primers listed in Table 1 and ligated into the plasmid pFBDM via restriction sites, and the fluorescent gene was simultaneously linked to another MCS of pFBDM. Consequently, the recombinant plasmids pFBDM-VSRs-egfp (consisting of pFBDM-p64-p0-egfp, pFBDM-p64-p19-egfp, pFBDM-p64-core-egfp, pFBDM-p10-p0-egfp, pFBDM-p10-p19-egfp and pFBDM-p10-core-egfp) were successfully generated. To assess the effects of the VSR gene on the early and late expression efficiency of exogenous genes, dual luciferase proteins driven by both p64 and p10 promoters were also incorporated into the plasmid pUCDM. Similarly, the mCherry fluorescent gene was introduced into the plasmid as a marker.

### 2.5. Construction of Recombinant MultiBac and Generation of Recombinant Baculovirus

Referring to the previously developed insect MultiBac multiple expression system and liposome-free transfection technology, MultiBac containing multiple target genes was directly used to infect insect Sf9 cells and produced high-titer recombinant baculoviruses. The specific experimental procedures were as follows: firstly, vsr genes (*p0*, *p19,* and *core*), luciferase gene (Firefly and Renilla luciferase), and fluorescence gene (*egfp* and *mCherry*) carried by recombinant plasmids pFBDM and pUCDM were simultaneously introduced into the MultiBac using mini-Tn7 and cre-loxP transposition technology. After antibiotic screening, blue-white plate screening, and specific gene PCR identification, six different recombinant MultiBacs were obtained: rBac-p64-p0-F-R, rBac-p64-p19-F-R, rBac-p64-core-F-R, rBac-p10-p0-F-R, rBac-p10-p19-F-R, and rBac-p10-core-F-R. The control group of MultiBac only contained the luciferase gene and fluorescence gene without vsr genes. Subsequently, the seven *E. coli* MultiBac strains were individually incubated with normal insect Sf9 cells, facilitating the entry of recombinant MultiBac into Sf9 cells to achieve target gene expression mediated by the infection protein Invasin. The recombinant MultiBac constructs in this study harbored *egfp* and *mCherry* genes, enabling indirect visualization of the construction process through intracellular fluorescence changes. Finally, upon observing no significant alteration in fluorescence intensity within Sf9 cells, cell supernatants were collected for subsequent reinfection of normal insect Sf9 cells to complete the passage of the recombinant baculovirus. The resulting recombinant virus present in the supernatants was stored at 4 °C.

### 2.6. The Expression of Target Genes Was Determined Using Western Blotting

The recombinant baculovirus was incubated with normal Sf9 insect cells at 28 °C for 48–96 h (Multiplicity of Infection, MOI = 1). Subsequently, the cells were washed with PBS and resuspended. The cell pellets were collected and lysed to release intracellular proteins. Firstly, protein separation was performed using 10% sodium dodecyl sulfate-polyacrylamide gel electrophoresis (120 V voltage), and electrophoresis was terminated when the bromophenol blue indicator reached the bottom of the separating gel. Next, the target protein was transferred onto a nitrocellulose membrane (Millipore, Darmstadt, Germany) while being blocked with 5% skim milk powder. Following overnight incubation at 4 °C with a murine primary antibody against 6×His-tag (diluted to a concentration of 1:3000; Lot.NO: AF5060; Beyotime, Shanghai, China), HRP-labeled murine secondary antibody IgG (diluted to a concentration of 1:4000; Thermo Fisher, Waltham, MA, USA) was added and incubated for two hours. Moreover, α-tubulin was employed as a reference protein in this study, and its identification was performed through Western blot analysis using rabbit α-tubulin antibody (diluted at a concentration of 1:3000; Lot.NO: AF5012; Beyotime, Shanghai, China). Finally, a chemical chromogenic solution was added and subjected to chromogenic imaging in an ECL chemiluminescence system.

### 2.7. Flow Cytometry to Determine Apoptosis in Insect Sf9 Cells

The apoptosis rate of insect Sf9 cells at different time points after virus infection was analyzed using the Annexin V-Elab Fluor^®^ 647/PI apoptosis detection kit (Elabscience, Wuhan, China) and flow cytometry. Annexin V is a calcium-dependent phospholipid-binding protein with a high affinity for phosphatidylserine. During cellular apoptosis, phosphatidylserine on the inner side of the membrane translocates to the outer surface, where it can bind to fluorescently labeled Annexin V (Elab Fluor^®^ 647), allowing detection by flow cytometry or fluorescence microscopy. Propidium iodide (PI) specifically binds to double-stranded DNA in late apoptotic or necrotic cells, resulting in strong fluorescence. When used in conjunction with Annexin V, PI can distinguish cells at different stages of apoptosis. To confirm successful viral infection, expression of eGFP protein was detected within 24–140 h after incubation of recombinant baculovirus and insect cells using inverted fluorescence microscopy. After centrifugation and collection of cell sediment at low temperature, the cells were washed with PBS and resuspended in 1 × Annexin V Binding Buffer. Sequential addition of 5 μL Annexin V-Elab Fluor^®^ 647 Reagent and 5 μL PI Reagent (50 μg/mL) followed by gentle swirling and incubation at room temperature for 15–20 min under light avoidance before analysis by flow cytometry. Normal Sf9 cells and wild-type virus-infected cells without variable short repeats factors were included as controls for flow compensation regulation to exclude any influence from wild-type virus infection on apoptosis.

### 2.8. Evaluation of the Recombinant Baculovirus Titers Using TCID_50_

The insect Sf9 cells in the logarithmic growth phase were evenly distributed into 96-well plates (with a cell density of approximately 10^5^ cells per well) and incubated at 28 °C overnight to ensure complete cell adhesion. Subsequently, the cell supernatant was discarded, and 100 μL of 10-fold concentrated recombinant baculovirus carrying fluorescent protein was added to each well. After incubating the recombinant baculoviruses with Sf9 cells at 28 °C for 4–6 h (MOI = 1), fresh insect cell medium was added to replace the previous medium and fluorescence expression was recorded using a fluorescence microscope after 72 h. The method of Reed and Muench’s standard was used to obtain the titer of recombinant baculovirus (TCID_50_) [34].

### 2.9. Statistical Analysis

The statistical analysis was performed using GraphPad Prism 7 software (Version.NO: Windows 7.05; GraphPad Software, Boston, MA, USA), and all values are presented as mean ± standard deviation (SD). Significant differences were determined using either one-way ANOVA or the Kruskal–Wallis test. A *p*-value of less than 0.05 was considered statistically significant.

## 3. Results

### 3.1. The Normal Cellular Functions Are Maintained in Insect Cells That Overexpress VSRs

**Figure 1:**
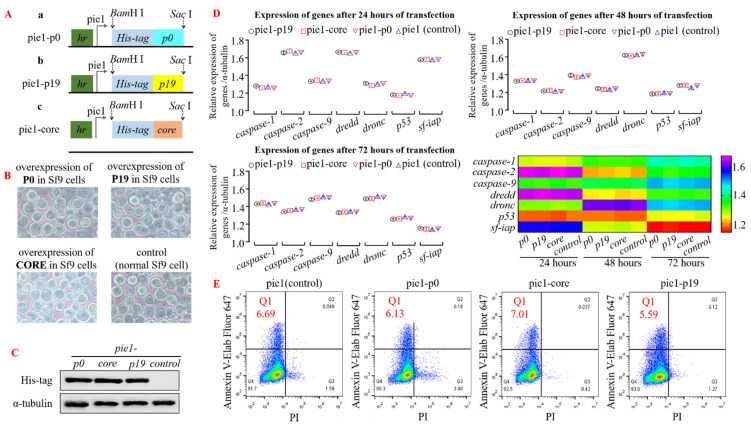
Insect Sf9 cells exhibit normal biological functions even when heterologous VSR is overexpressed. (**A**) The schematic diagram illustrates the construction of transient expression plasmids: (a) Plasmid pie1-p0, (b) Plasmid pie1-p19, and (c) Plasmid pie1-core. (**B**) Transfection of recombinant plasmids into Insect Sf9 cells for 48 h does not induce any morphological changes in their structure. (**C**) Western blot analysis using His-tag antibody confirms successful overexpression of VSR protein in Sf9 cells (48 h post-transfection), with α-tubulin serving as a control. (**D**) qPCR results reveal no significant alteration in the expression levels of apoptotic genes in normal insect Sf9 cells after 24–72 h of VSR protein overexpression. (**E**) Flow cytometry analysis demonstrates that the apoptosis rate of Sf9 cells remains unchanged across different groups following VSR protein overexpression (48 h post-transfection; Annexin V-647 and Propidium Iodide used as fluorescent dyes).

To investigate the impact of heterologous viral suppressor of RNA silencing (VSR) on insect cells or intracellular RNA interference (RNAi) mechanism, we initially assessed gene regulation and cell cycle in insect cells upon transfection with a plasmid overexpressing VSR. The four transient expression plasmids, pie1-p0, pie1-p19, pie1-core, and pie1 (control group), were transfected into insect Sf9 cells (Figure 1A,B). Subsequently, Western blot analysis was performed using the His-tag antibody to detect protein expression in the cell precipitates of each group after incubation at 28 °C for 24 h. The results demonstrated successful high-efficiency expression of P0, P19, and CORE proteins in Sf9 cells (Figure 1C; α-tubulin served as an internal reference). To investigate the impact of VSR expression on insect cells further, total RNA was extracted from the transfected Sf9 cells after 24–72 h of incubation for reverse transcription. The qPCR analysis revealed no significant changes in the expression levels of apoptosis-related genes (such as *caspase* and *iap*) compared to the control group, indicating that overexpression of VSR derived from single-stranded RNA viruses did not affect the insect Sf9 cells (Figure 1D). Additionally, flow cytometry was employed to measure the apoptosis rate of transfected Sf9 cells after 48 h. However, no significant differences were observed among the cell groups expressing VSR derived from plant or animal viruses with regard to gene expression regulation and cell cycle modulation (Figure 1E). Consequently, our findings demonstrate that the overexpression of RNA interference inhibitors in insect Sf9 cells does not exert any discernible impact on the apoptotic progression of these normal cells.

### 3.2. The Presence of VSRs Markedly Attenuated the Impact of Intracellular RNA Interference

**Figure 2:**
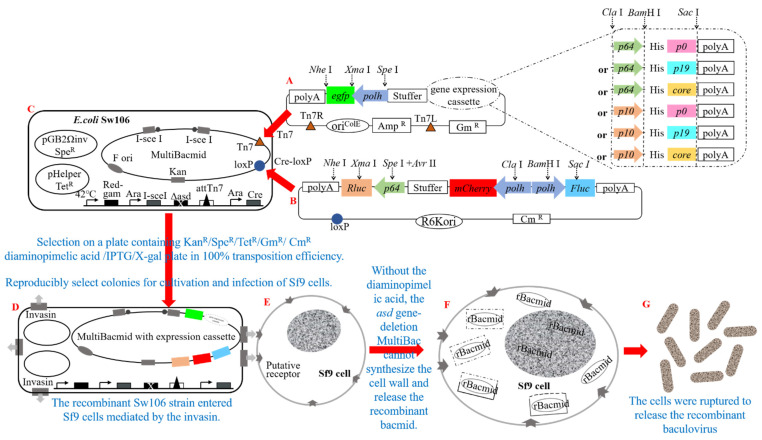
Schematic representation illustrating the generation of a recombinant baculovirus harboring the vsr gene, luciferase gene, and fluorescent protein gene. (**A**) Schematic diagram depicting the construction of recombinant plasmids pFBDM-vsr-polh-egfp driven by various promoters (vsr: p64-p0, p64-p19, p64-core, p10-p0, p10-p19, p10-core). (**B**) Schematic diagram outlining the assembly of recombinant plasmid pUCDM-p64-Rluc-polh-Fluc-polh-mCherry containing luciferase genes. (**C**) Illustration presenting *E. coli* Sw106 strain hosting MultiBacmid. (**D**) Diagram demonstrating the integration of the vsr gene, luciferase gene, and fluorescent protein gene into recombinant *E. coli* Sw106 MultiBacmid using Tn7 and Cre-loxP transposition technology. (**E**) Representation showcasing normal insect Sf9 cells. (**F**) Successful invasion of Sf9 cells by recombinant *E. coli* Sw106 facilitated by Invasin, resulting in rBacmid release. (**G**) Schematic diagram displaying the construction of high-expression recombinant baculoviruses carrying different vsr genes, luciferase genes, and fluorescent protein genes.

In this study, our objective was to induce RNAi in insect Sf9 cells through viral infection and subsequently investigate the impact of heterologous VSR on this intricate interference mechanism. To accomplish this, we successfully generated seven recombinant baculoviruses using specific transposition techniques (Tn7 and Cre-loxP), the MultiBac system, and lipofectamine-free transfection following Section 2.4 and Section 2.5 (Figure 2).

**Figure 3:**
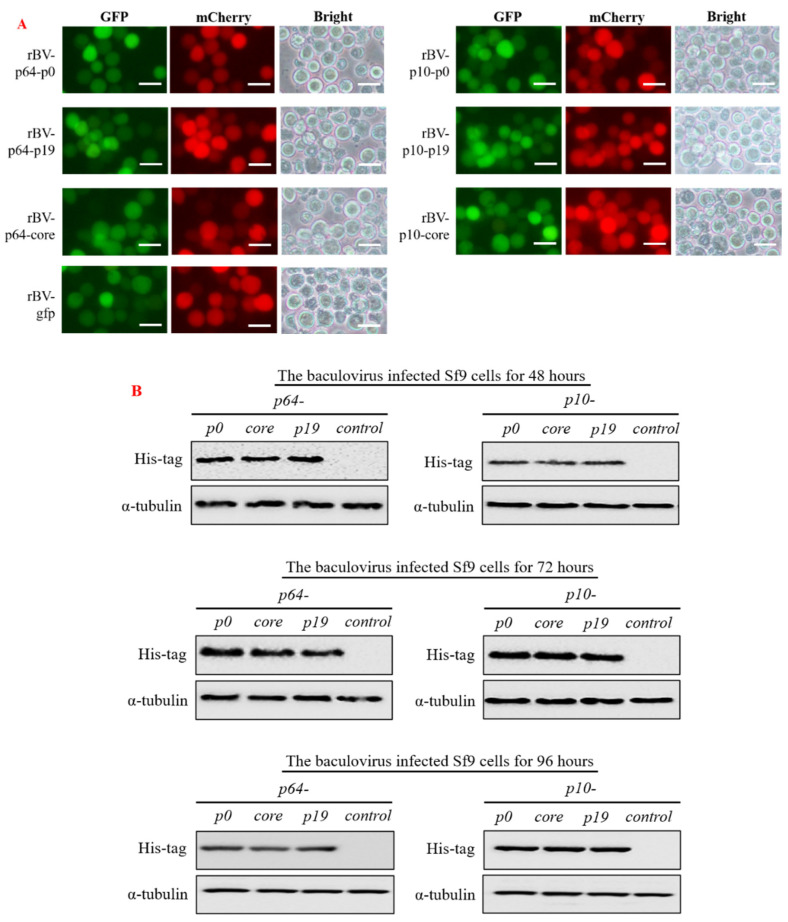
The recombinant baculovirus carrying the target gene successfully infected insect Sf9 cells, and the expression of VSR protein in the cells was identified using Western blot. (**A**) After incubation of the recombinant baculovirus with normal insect Sf9 cells for 48 h (MOI = 1), fluorescence microscopy revealed the presence of both green and red fluorescence signals within the insect Sf9 cells, confirming successful infection by the recombinant virus and expression of the target protein (Bar = 50 µm). (**B**) After infecting insect Sf9 cells with the recombinant baculovirus, cell pellets were collected at various time points during infection. Subsequently, intracellular proteins were identified using Western blot analysis with His-tag antibody and α-tubulin antibody. The results demonstrated a significant upregulation of VSR in Sf9 cells between 48- and 96 h post-infection, with the highest expression level observed at 72 h post-infection. All the Western blot experiments were conducted as parallel experiments, and gel blots were processed simultaneously (Supplementary Materials, Figure S1).

Following a 48 h incubation period with the recombinant baculovirus (MOI = 1), we effectively visualized high levels of red and green fluorescent proteins in Sf9 cells using fluorescence microscopy, confirming successful infection by the recombinant baculovirus (Figure 3A). The subsequent observation revealed that Sf9 cells in different treatment groups exhibited no discernible cytopathic effect or rupture compared to the control group (rBV-egfp). Additionally, we utilized antibodies against His-tag and α-tubulin protein (as a reference) for Western blot analysis to identify precipitates from Sf9 cells after incubation with the virus for 48, 72, and 96 h, respectively. The results demonstrated robust expression of VSR proteins in Sf9 cells (Figure 3B).

**Figure 4:**
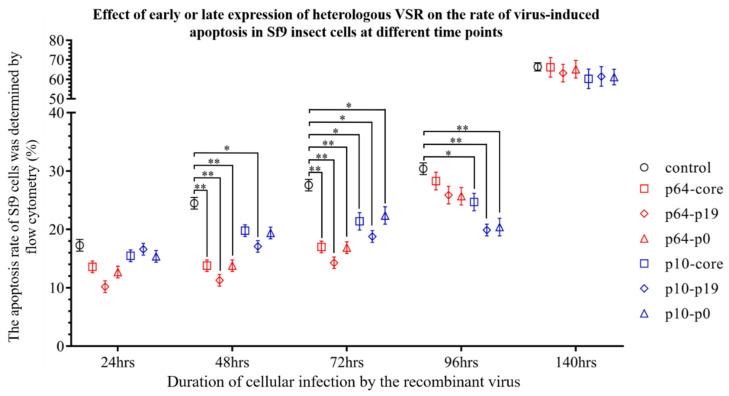
Impact of early or later expression of heterologous VSR on the rate of virus-induced apoptosis in Sf9 insect cells at distinct time intervals. (*: *p* < 0.05; **: *p* < 0.01; Compared with the control group).

Subsequently, flow cytometry was employed to investigate the impact of early and late expression of VSRs on the apoptosis rate of virus-induced insect Sf9 cells (Figure 4). To eliminate the influence of wild-type baculovirus-induced apoptosis and programmed cell death in this study, normal Sf9 cells and wild-type baculovirus-infected Sf9 cells were utilized as controls. The findings demonstrated that VSRs significantly suppressed virus-induced apoptosis within 24–96 h post-virus infection. Specifically, the early promoter p64 exhibited a more pronounced inhibitory effect on Sf9 cell apoptosis within 24–48 h compared to p10 (*p* < 0.05), while the inhibitory effect of p19 protein surpassed that of p0 and core protein. On the other hand, the late promoter p10 initiated its anti-apoptotic activity 48 h after virus infection and attained maximum inhibition efficacy within 72–96 h (*p* < 0.05), with the p19 protein demonstrating superior effectiveness. These results indicate that both early and late expression of VSR exerts varying degrees of influence on virus-induced apoptosis at different time intervals. Notably, evident apoptosis occurred in Sf9 cells, including those in the control group, after 140 h post-virus infection (*p* > 0.05), which further confirms that VSR primarily inhibits virus RNAi during early-stage apoptosis but has no impact on late-stage apoptosis or necrotic cells (The detailed statistics of apoptosis rates are provided in Supplementary Materials, Figure S2). In summary, the heterologous VSR significantly prolonged the life cycle of infected insect Sf9 cells and reduced the apoptosis rate, as compared to the control group.

**Figure 5:**
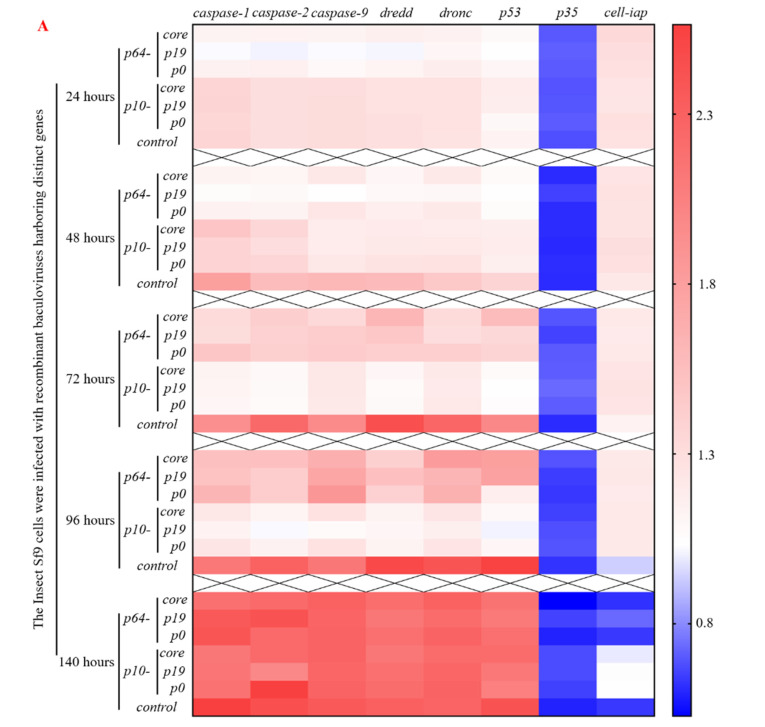

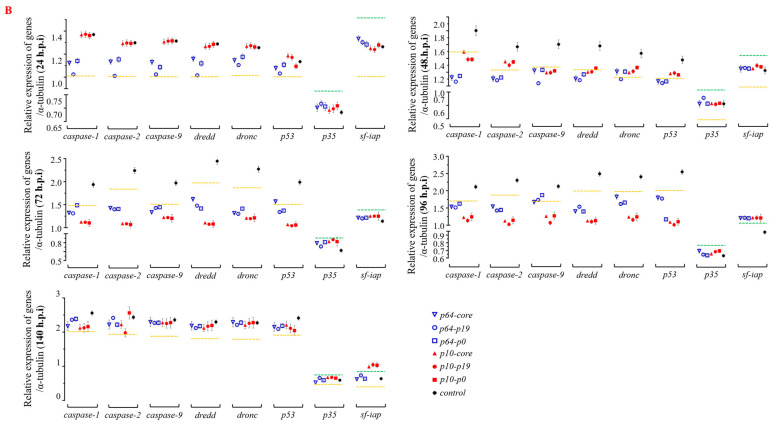
Repercussions of heterologous VSR on the regulation of apoptotic gene expression in virus-infected host Sf9 cells. (**A**) Heat map illustrating the impact of heterologous VSR on the regulation of apoptotic gene expression in Sf9 cells during viral infection, depicting early and late expression patterns. (**B**) Significance analysis of differential expression levels of host apoptotic genes at different time points during viral infection, with values above the green dotted line or below the yellow dotted line indicating statistical significance (*p* < 0.05).

To further elucidate the molecular mechanism underlying the delay of apoptosis by heterologous VSR, the recombinant viruses from the seven groups were reintroduced into normal Sf9 cells. Total RNA was extracted and reverse transcribed within 24–140 h, followed by qPCR analysis to determine the expression of apoptosis genes such as *caspase* (Figure 5). The results demonstrated that within 24 h post-virus infection, the early promoter p64-driven VSR expression inhibited the expression of apoptosis genes *caspase-1*, *caspase-2*, *caspase-9*, *dredd*, *dronc*, and *p53* to varying degrees. Moreover, it was observed that the inhibition effect of P19 protein on apoptosis gene expression was superior to that of P0 and CORE proteins. Notably, there were no significant changes in the expression of apoptosis inhibitory genes *p35* and *sf-iap* compared to the control group. After 48 h of virus infection, a reduction in pro-apoptotic gene expression was observed in Sf9 cells compared to the control group. Particularly noteworthy was a significant downregulation (*p* < 0.05) in the expression levels of *caspase-1*, *caspase-2*, *caspase-9*, and *dredd* genes. Furthermore, the inhibition effect exerted by early promoter p64 remained superior to that exerted by p10 at this stage. Within a timeframe ranging from 72 to 96 h post-virus infection, the most pronounced inhibition effect on apoptosis gene expression was achieved by VSR (*p* < 0.05), while late promoter p10’s inhibition effect continued increasing, surpassing that exerted by p64. These findings demonstrate that VSR expression during the early and late stages of virus infection can influence the regulation and expression of apoptosis genes, with varying degrees of inhibition observed at different time points. Furthermore, after 96 h post-infection with the recombinant baculovirus in Sf9 cells, VSR also promoted the expression of the anti-apoptotic gene *sf-iap* (*p* < 0.05). Although *caspase* gene expression increased after 140 h post-infection, there was no significant difference compared to the control group (*p* > 0.05), suggesting that this may be attributed to VSR primarily inhibiting viral RNA interference mechanisms rather than affecting late-stage apoptosis or cell necrosis in Sf9 cells. Therefore, based on the molecular mechanism of apoptosis inhibition, the introduction of heterologous VSR into insect Sf9 cells significantly attenuated the expression of apoptotic genes, thereby impeding apoptosis progression.

### 3.3. The Presence of VSR Significantly Enhanced the Expression Efficiency of the Recombinant Protein in the MultiBac System

**Figure 6:**
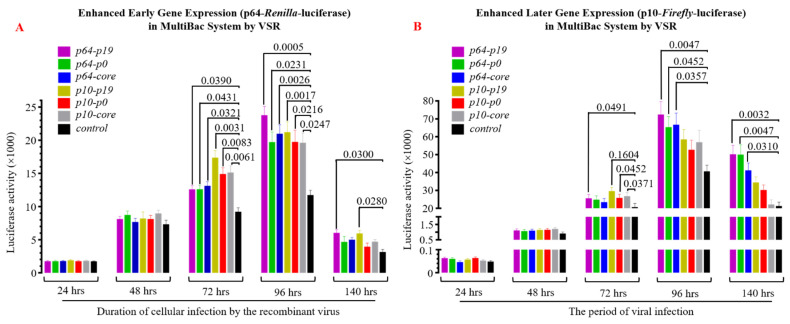
Impact of heterologous VSR on protein expression efficiency in insect cell bioreactors. (**A**) The expression of early protein Renilla-luciferase in insect Sf9 cells was significantly enhanced by heterologous VSR. (**B**) The expression of late protein Firefly-luciferase in insect Sf9 cells was significantly increased upon the introduction of heterologous VSR.

To investigate whether VSR enhances the expression efficiency of the recombinant baculovirus in insect cells, we introduced two luciferase reporter genes (Renilla- and Firefly-luciferase) into the viral genome using MultiBac in the recombinant baculovirus construction. The early promoter p64 and late promoter p10 of the virus were employed to drive luciferase expression separately. Analysis of luciferase detection results revealed that VSR could also enhance the efficiency of target gene expression to varying degrees in Sf9 cells 48 h after viral gene expression initiation. Specifically, within 72–96 h post-viral infection (MOI = 1), the expression of early protein (Renilla Luciferase, Figure 6A) was significantly higher than that observed in the control group (*p* < 0.05), while significant differences were observed for late protein (Firefly Luciferase, Figure 6B) within 96–140 h (*p* < 0.05). Notably, the P19 protein exhibited a more pronounced effect on enhancing exogenous gene expression than P0 and CORE. These findings provide initial evidence confirming that VSR derived from single-stranded RNA viruses also possesses the ability to improve exogenous protein expression efficiency in baculovirus expression systems, thereby augmenting the applicability of insect bioreactors.

### 3.4. The Presence of VSRs Enhance the Production of Recombinant Viruses with Higher Titers in the MultiBac System

**Figure 7:**
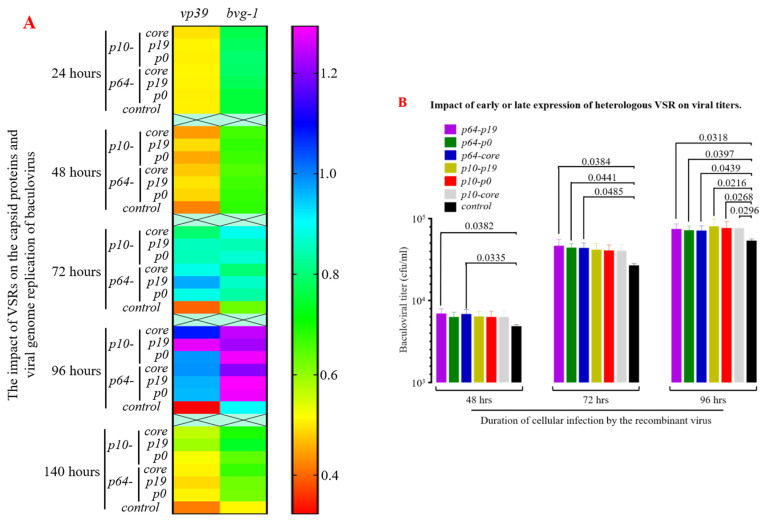
Impact of efficient heterologous VSR expression on viral nucleocapsid replication and progeny viral titers. (**A**) Heterologous VSR significantly upregulated the expression of capsid protein VP39 and enhanced the replication efficiency of the baculovirus genome. (**B**) Heterologous VSR markedly augmented the titer of baculovirus production in an insect cell bioreactor.

The *vp39* sequence serves as the primary structural protein gene of the insect baculovirus capsid, while the *bvg-1* sequence represents the specific core region of the baculovirus. Both sequences are highly conserved and commonly employed to assess progeny virus proliferation and replication rates. Reverse transcription and qPCR analysis of total RNA from infected Sf9 cells (Figure 7A) revealed a significant increase in expression of *vp39* and baculovirus genes after 48 h post-viral infection (MOI = 1), with maximum significant differences observed at 72–96 h compared to the control group (*p* < 0.05). Additionally, using the TCID_50_ method to measure progeny virus titers demonstrated that VSR enhanced nuclear capsid assembly efficiency to varying degrees within 48–96 h after viral infection (MOI = 1), particularly during 72–96 h when progeny virus titers were significantly higher than those in the control group (*p* < 0.05, Figure 7B). Simultaneously, VSR strongly suppresses apoptosis in Sf9 cells within 72–96 h post-viral infection, providing “cell factories” with extended time for gene replication, protein expression, and nuclear capsid assembly. Furthermore, the determination of progeny virus titers also indicated that P19 exhibited significantly superior effects compared to P0 and CORE proteins. These results demonstrate that the incorporation of heterologous VSR can significantly enhance the yield of recombinant viruses in the MultiBac system, thereby holding significant implications in biological pesticides and viral vaccine vectors.

## 4. Discussion

RNAi is a highly conserved post-transcriptional regulatory mechanism that is ubiquitously present in eukaryotes. Biological cells employ this intricate defense strategy to counteract viral invasion [35,36]. In response, viruses have evolved sophisticated RNAi inhibitors to subvert this host defense mechanism and ensure their transcription and replication [37,38]. These inhibitors play pivotal roles as virulence factors, primarily facilitating viral invasion, replication, and interference with host cellular processes [39,40]. Numerous RNAi inhibitors have been identified from plant and animal viruses [41,42,43].

VSRs used in this study were derived from single-stranded RNA viruses infecting plants or animals and exhibited significant inhibitory effects on virus-induced apoptosis in insect cells. Molecular analysis of early apoptosis revealed that during the initial 24 h of viral infection in Sf9 cells, the expression of apoptosis-related genes was interfered with and inhibited. This suggests the presence of VSRs binding to double-stranded RNA molecules at an early stage of the RNAi process, preventing their cleavage. It also indicates that VSRs in insect cells share characteristics similar to those found in plant or animal cells, such as the P19 protein’s ability to bind with newly formed 21 nt and 22 nt RNA in plant cells, thereby hindering RISC assembly [26,44]. This regulatory mechanism is not limited to single-stranded RNA viruses but is also observed with FHV-B2 protein, NS1 protein of influenza A virus, Tomato aspermy virus (TAV)-2b protein, and other similar proteins [45,46,47]. Despite changes in gene regulation, there was no significant alteration observed within 24 h regarding the rate of apoptosis in Sf9 cells. This suggests that early viral infection does not initiate obvious apoptotic pathways immediately; there may be a certain lag between molecular regulation of apoptosis-related genes and the actual occurrence of apoptosis [48]. During the middle stage (48–96 h) of viral infection, both expression levels of apoptotic genes and cell death rates changed significantly. The effects varied depending on different time periods for p64 promoter and p10 promoter activities, mainly influenced by VSR expression levels during early and late stages. Combined with previous analysis on promoter efficiency, it was demonstrated that p64 activity primarily occurred within 12–24 h after viral infection, while p10 showed exceptionally high expression efficiency during 48–96 h [49]. The results demonstrated a significant increase in apoptosis at 140 h post-virus infection in all experimental groups, indicating the exclusive efficacy of VSRs against the RNAi mechanism while exerting no discernible impact on cells that had already undergone extensive apoptosis.

Additionally, a dose effect of viral suppressor of RNA silencing (VSR) has been observed in plant cells and viruses, where high titer virus infection can significantly disrupt the silencing effect of VSR on RNAi [50]. Therefore, we hypothesize that this phenomenon also exists between insect cells and viruses. Previous studies have demonstrated that virus MOI greatly impacts virus proliferation ability and protein expression [51,52]. For instance, a higher yield of recombinant baculovirus and non-secreted proteins can be obtained using insect High Five and Sf21 cells as bioreactors with an MOI between 1–3 and incubation time between 72–96 h. However, a lower MOI (0.1–0.5) is generally used for secreted protein production to ensure host cell viability post-infection. When MOI > 5 is applied, intracellular progeny virus replication is significantly inhibited by density inhibition effects, leading to rapid host cell rupture. The rapid cell rupture results in reduced virus titer and protein expression levels. In our study, we utilized an MOI = 1 to synchronize all infected cells while minimizing the impact of rapid cell death caused by high MOIs.

Previous studies have demonstrated that the virus’s replication relies on host cells, making the double-stranded RNA replication intermediate a prime target for RNAi attacks. Despite being derived from single-stranded RNA viruses, P19, P0, and CORE proteins exhibit non-conservative core sequences and employ distinct pathways to inhibit RNAi [53]. Compared to P0 and CORE proteins, the P19 protein exhibits superior efficacy in regulating apoptotic gene expression and inhibiting apoptosis in insect cells. Based on existing research reports, it is speculated that the P19 protein not only directly binds to dsRNA but also interacts with siRNA to impede complex assembly and formation [54]. This multifaceted involvement in various processes of RNAi inhibition enhances its VSR function. Furthermore, studies have indicated that the P19 protein substantially reduces free 21–22 nt siRNAs within plant nuclei while having no effect on cytoplasmic siRNA levels [55,56]. These findings suggest that P19 primarily acts as an early protein inhibitor of RNA interference within the plant nucleus. The nucleopolyhedrovirus carrying the VSR gene used in this study completes gene transcription and viral replication predominantly within the nucleus [57], which may explain why the application of the P19 protein yielded notably better results than the P0 protein and CORE protein. Furthermore, we will explore the utilization of the MultiBac system to achieve early and late co-expression of VSR with diverse modes of action. Additionally, we aim to investigate the synergistic effect of VSR through simultaneous inhibition of multiple pathways, including dsRNA cleavage, siRNA synthesis, and interference complex assembly, to further enhance the longevity of recombinant virus within host cells, thereby enhancing the potential application prospects in insect cell bioreactors.

The recombinant baculovirus genome constructed in this study carries the VSR gene and harbors two luciferase genes expressed during early and late stages. The data demonstrated a significant increase in mRNA levels of the two luciferase genes within 72 h post-virus infection, indicating that VSR enhances the transcription efficiency of the target gene by counteracting RNAi and thereby augmenting target protein expression. Similar findings were observed for capsid protein VP39 expression in subsequent generations of infected cells (Figure 6A). Amplification of the baculovirus genome revealed that there was no substantial increase in baculovirus gene content within 48 h after virus infection, primarily due to the absence of RNA interference-mediated inhibition on the DNA replication process. The subsequent rise in late baculovirus genome replication and progeny virus titer was speculated to be mainly attributed to delayed apoptosis, which provided more time for DNA replication and nuclear capsid assembly. Therefore, investigating the impact of VSR on insect cells and viruses through recombinant baculovirus vectors not only elucidates the antiviral mechanisms employed by insect cells but also enhances the potential application value of insect cell bioreactors in high-titer recombinant baculovirus production and efficient expression of recombinant proteins via targeted construction of stable cell lines.

## 5. Conclusions

In conclusion, RNA interference silencing factors derived from plant and mammalian viruses exhibit significant inhibitory effects on the RNA interference phenomenon in insect Sf9 cells. The most pronounced inhibition occurs within 48–72 h of viral infection through binding to dsRNA. Moreover, incorporating these inhibitors into the protein expression system based on insect cells can greatly enhance recombinant protein expression and production titer of recombinant viruses, thereby expanding the application range and value of insect bioreactors.

## Figures and Tables

**Table 1:** Primers used to amplify target genes.

Name	Sequence	Genes
*p0* (with His-tag)	F: 5′-cccgggatgcatcatcaccaccatcacatgattgtattgacccagt R: 5′-gcatgcttattcttgtaattccttt	Potato leafroll virus p0 Gene
*p19* (with His-tag)	F: 5′-cccgggatgcatcatcaccaccatcacatggaacgagctatacaag R: 5′-gcatgcttactcgctttctttttcg	Tomato bushy stunt virus p19 Gene
*core* (with His-tag)	F: 5′-cccgggatgcatcatcaccaccatcacatgagcacgaatcctaaac R: 5′-gcatgcttaagccgaggcgggaacg	*Hepacivirus C* core Gene
*Fluc*	F: 5′-ggatccatggaagacgccaaaaacata R: 5′-tctagattacacggcgatctttccg	Firefly-luciferase Gene
*Rluc*	F: 5′-cccgggatgacttcgaaagtttatgat R: 5′-gcatgcttattgttcatttttgagaact	Renilla-luciferase Gene
*egfp*	F: 5′-ggatccatggtgagcaagggcgagga R: 5′-tctagattacttgtacagctcgtccat	Enhanced Green Fluorescent Protein Gene
*mCherry*	F: 5′-cccgggatggtgagcaagggcgagga R: 5′-gctagcttacttgtacagctcgtccatgcc	mCherry Fluorescent Protein Gene

The 5′ end of the primer design corresponds to the sequence of the restriction enzyme site (underlined), while the shaded region represents the His-tag sequence.

**Table 2:** Primers used for qPCR.

Name	Sequence	Genes
*α-Tubulin*	F: 5′-agtccagatcggtaatgc R: 5′-gctgaagaaggtgttgaag	Reference Gene
*bvg-1*	F: 5′-cccgtaacggacctcgtactt R: 5′-ttatcgagatttatttgcatacaac	Baculovirus Genome
*Caspase*-1	F: 5′-gattcaaagttacggtgttcccta R: 5′-ggttgtctggcttgtaatgagtat	Cysteine Aspartate-specific Gene
*Caspase-2*	F: 5′-gtaaggttctgattggcaattagc R: 5′-cggtacttgtggttggtgtt	Cysteine Aspartate-specific Gene
*Caspase*-9	F: 5′-acacagagtttgacaacaatatcg R: 5′-ggtctcatagtccaccaacac	Cysteine Aspartate-specific Gene
*Dronc*	F: 5′-ctggtagatacgcttggagaacta R: 5′-gcctgtttgatgtgctaagact	Apoptosis Gene
*Dredd*	F: 5′-aacaccacaaggaatggaagt R: 5′-agttacaggcatcgttggaa	Apoptosis Gene
Sf9-*iap*	F: 5′-gttggagagttgtgttgtttgttt R: 5′-aatagcgttaatgttgaggaggag	Inhibitor of Apoptosis Gene
*vp39*	F: 5′-acccgataagaagcagtgac R: 5′-cccagagtagcgttaggatt	Baculovirus Capsid Gene
*p35*	F: 5′-cgaacgcaacgactactac R: 5′-tgagcaaacggcacaataac	Apoptosis Gene
*p53*	F: 5′-caccgtctcaaccgtatc R: 5′-gaggacattcttcgctattt	Apoptosis Gene

## Data Availability

Most of the analytical data are presented in this article. Additional original datasets utilized and/or analyzed during the current study can be obtained from the corresponding author upon reasonable request.

## Supplementary Materials

The following supporting information can be downloaded at: https://www.mdpi.com/article/10.3390/insects15060375/s1, Figure S1: The Western blot Original Images in the manuscript (for Figure 1C and Figure 3B in the manuscript); Figure S2: The impact of RNA interference inhibitors on virus-induced apoptosis was evaluated at various time points using flow cytometry (for Figure 4 in the manuscript).
