# Supplementary material for: Impact of the Transboundary Interference Inhibitor on RNAi and the Baculovirus Expression System in Insect Cells
Source: Insects. 2024 May 21;15(6):375. doi: 10.3390/insects15060375 (PMC11203448; doi:10.3390/insects15060375)
Supplement: Supplementary file 1 [file insects-15-00375-s001.zip › insects-2980370-supplementary.pdf]

Figure 1S. The Western blot Original Images in the manuscript (for Figure 1.C and Figure3.B in the manuscript).

A: The successful expression of VSR in transfected Sf9 cells was confirmed by Western blot analysis using a His-tag antibody (for Figure 1.C in the manuscript).

B: Western blot analysis of transfected Sf9 cells was performed using an α-tubulin antibody, with α-tubulin serving as the internal control protein (for Figure 1.C in the manuscript).

C: The recombinant baculovirus was employed to infect insect Sf9 cells 48 days post-infection, and the cellular precipitation was detected via Western blot analysis using a His-tag antibody. The findings demonstrated successful expression of VSR facilitated by different promoters (for Figure 3.B in the manuscript).

D: After infecting Sf9 insect cells with recombinant baculovirus for 48-72 hours, Western blot analysis was performed using α-tubulin antibody as a reference protein to identify cell precipitates expressing VSR under the control of the p64 promoter (for Figure 3.B in the manuscript).

a: Cells were infected with the baculovirus for 72 hours.

b: Cells were infected with the baculovirus for 48 hours.

E: After infecting Sf9 insect cells with recombinant baculovirus for 72 days, cell precipitation was detected through Western blot analysis using a His-tag antibody. The results demonstrated successful expression of VSR mediated by different promoters (for Figure 3.B in the manuscript).

F: After infecting Sf9 insect cells with recombinant baculovirus for 48-72 hours, Western blot analysis was performed using α-tubulin antibody as a reference protein to detect cell precipitates expressing VSR under the control of the p10 promoter (for Figure 3.B in the manuscript).

a: Cells were infected with the baculovirus for 48 hours.

b: Cells were infected with the baculovirus for 72 hours.

G: After infecting Sf9 insect cells with recombinant baculovirus for 96 days, cell precipitation was detected through Western blot analysis using a His-tag antibody. The results demonstrated successful expression of VSR mediated by different promoters (for Figure 3.B in the manuscript).

H: At 96 hours post-infection of Sf9 insect cells with recombinant baculovirus, cell precipitation from different treatment groups was detected via Western blot analysis using an α-tubulin antibody as an internal reference protein (for Figure 3.B in the manuscript).

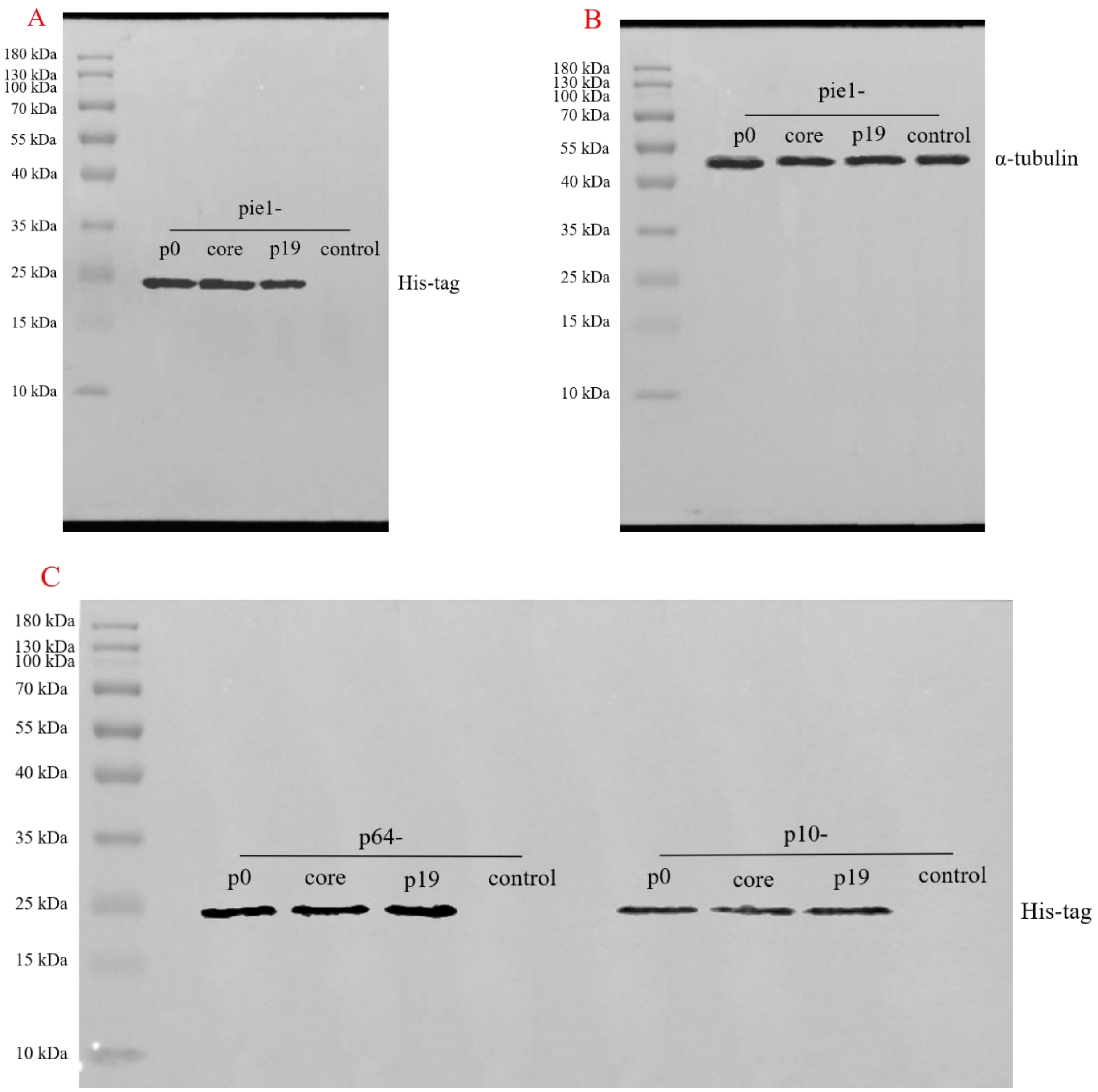
A
180 kDa
130 kDa
100 kDa
70 kDa
55 kDa
40 kDa
35 kDa
25 kDa
15 kDa
10 kDa
pie1-
p0
core
p19
control
His-tag
B
180 kDa
130 kDa
100 kDa
70 kDa
55 kDa
40 kDa
35 kDa
25 kDa
15 kDa
10 kDa
pie1-
p0
core
p19
control
α-tubulin
C
180 kDa
130 kDa
100 kDa
70 kDa
55 kDa
40 kDa
35 kDa
25 kDa
15 kDa
10 kDa
p64-
p0
core
p19
control
p10-
p0
core
p19
control
His-tag

D

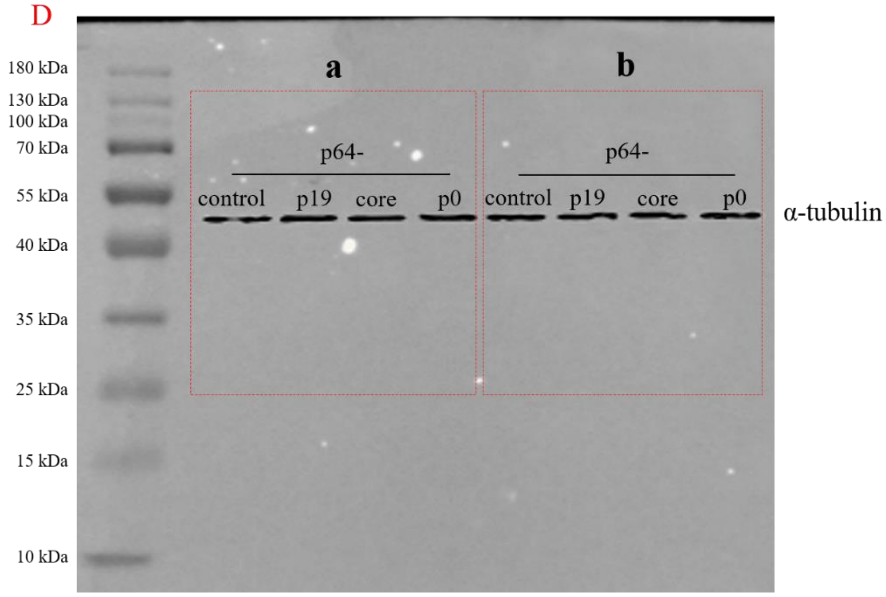
180 kDa
130 kDa
100 kDa
70 kDa
55 kDa
40 kDa
35 kDa
25 kDa
15 kDa
10 kDa
a
b
p64-
p64-
control
p19
core
p0
control
p19
core
p0
α-tubulin

E

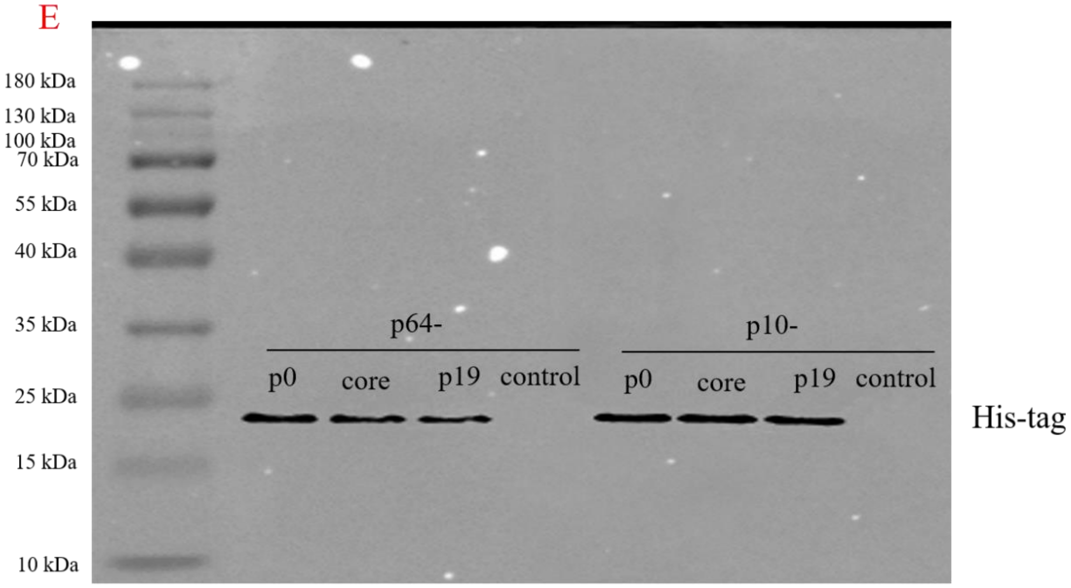
180 kDa
130 kDa
100 kDa
70 kDa
55 kDa
40 kDa
35 kDa
25 kDa
15 kDa
10 kDa
p64-
p10-
p0
core
p19
control
p0
core
p19
control
His-tag

F

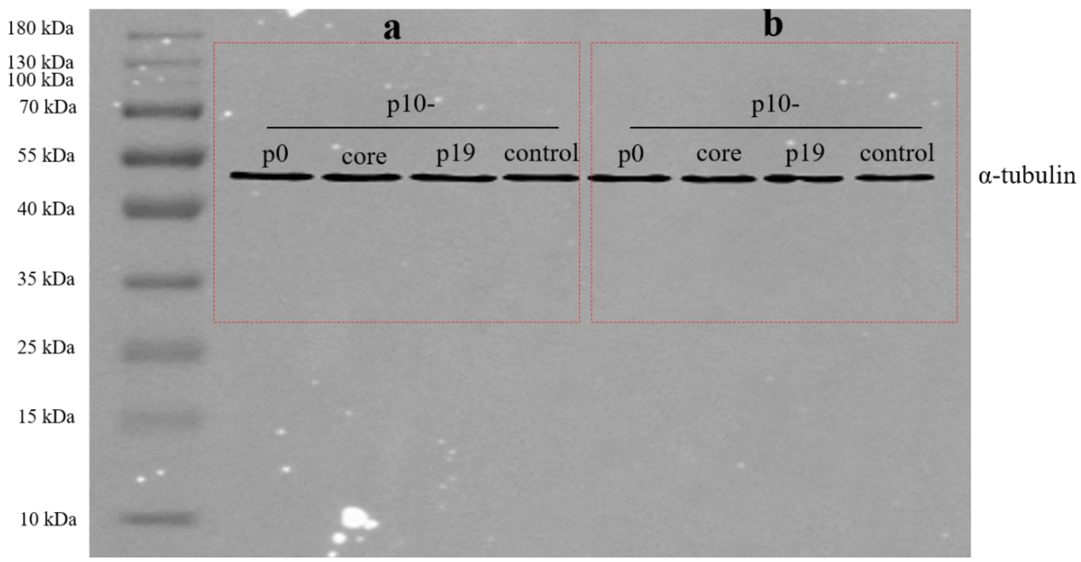
180 kDa
130 kDa
100 kDa
70 kDa
55 kDa
40 kDa
35 kDa
25 kDa
15 kDa
10 kDa
a
b
p10-
p10-
p0
core
p19
control
p0
core
p19
control
α-tubulin

G

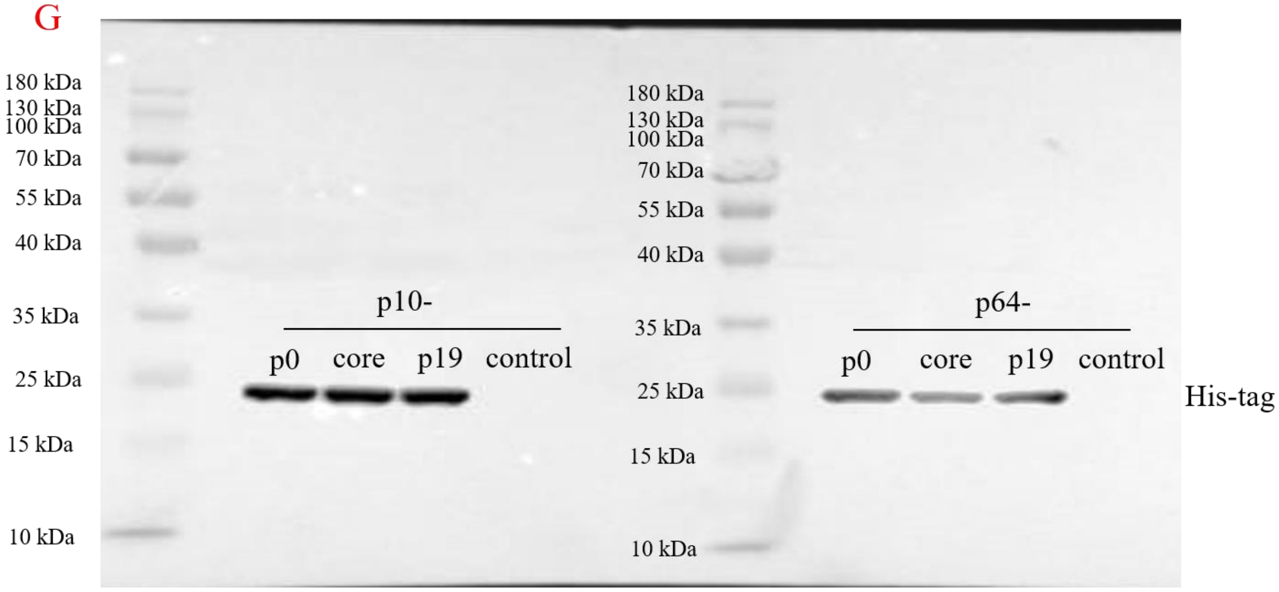
180 kDa
130 kDa
100 kDa
70 kDa
55 kDa
40 kDa
35 kDa
25 kDa
15 kDa
10 kDa
p10-
p0
core
p19
control
180 kDa
130 kDa
100 kDa
70 kDa
55 kDa
40 kDa
35 kDa
25 kDa
15 kDa
10 kDa
p64-
p0
core
p19
control
His-tag

H

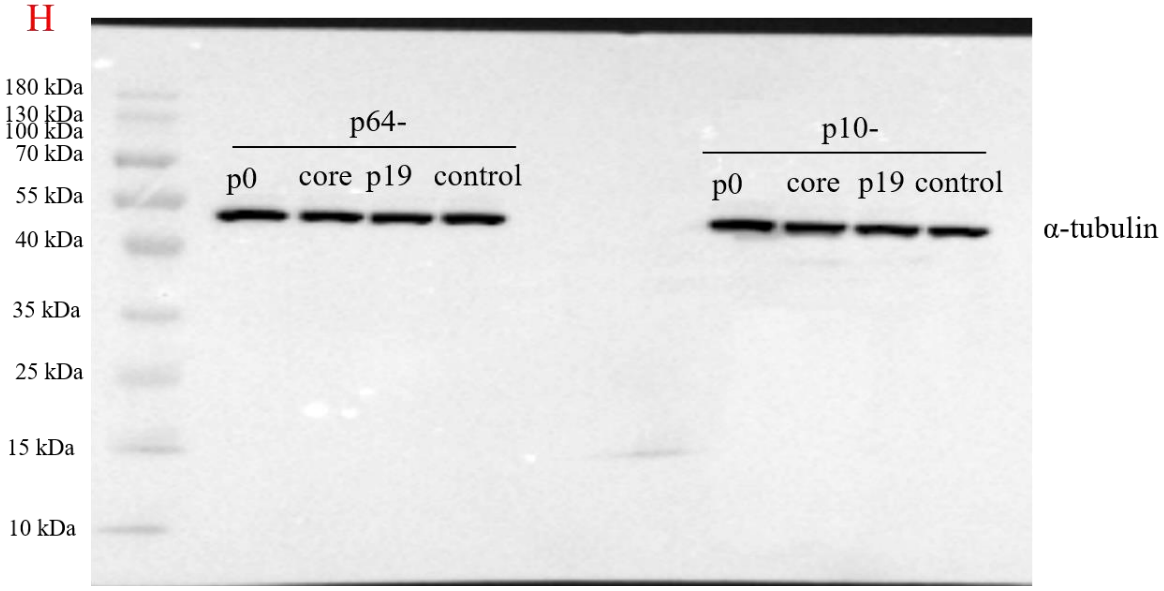
180 kDa
130 kDa
100 kDa
70 kDa
55 kDa
40 kDa
35 kDa
25 kDa
15 kDa
10 kDa
p64-
p0
core
p19
control
p10-
p0
core
p19
control
α-tubulin

Figure 2S. The impact of RNA interference inhibitors on virus-induced apoptosis was evaluated at various time points using flow cytometry (for Figure 4 in the manuscript).

The insect Sf9 cells were infected with recombinant baculovirus for a duration of 24-140 hours (**A**: 24 hours; **B**: 48 hours; **C**: 72 hours; **D**: 96 hours; **E**: 140 hours), and subsequently, the apoptosis rate of the cells was determined using flow cytometry.

A

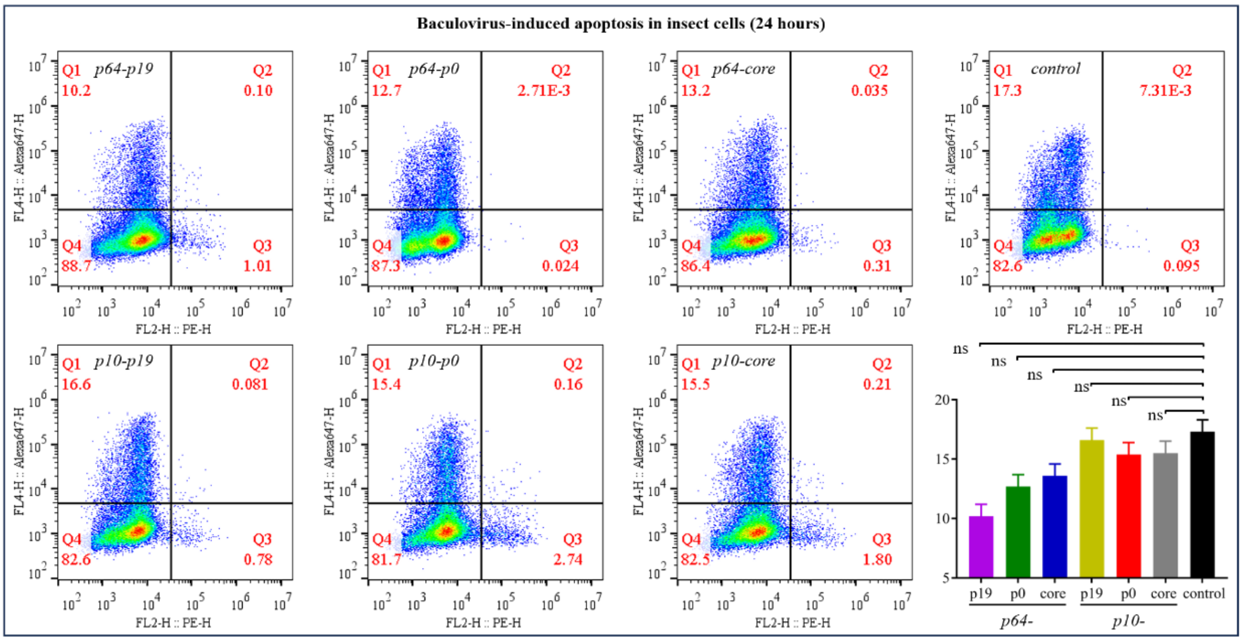

B

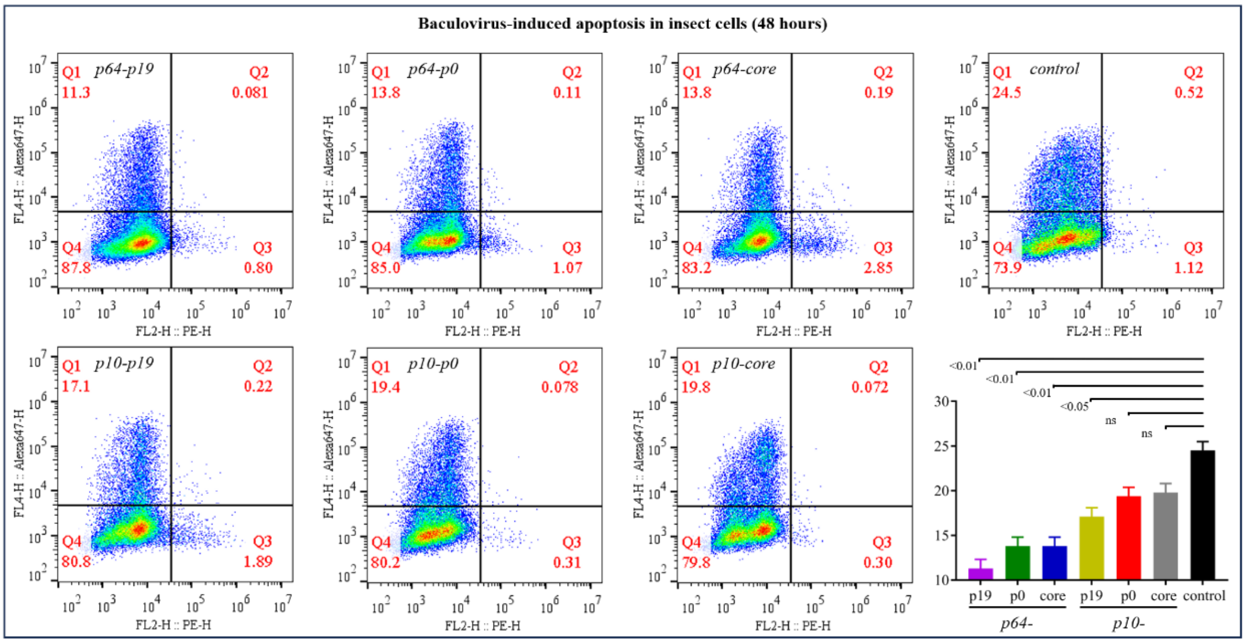

C

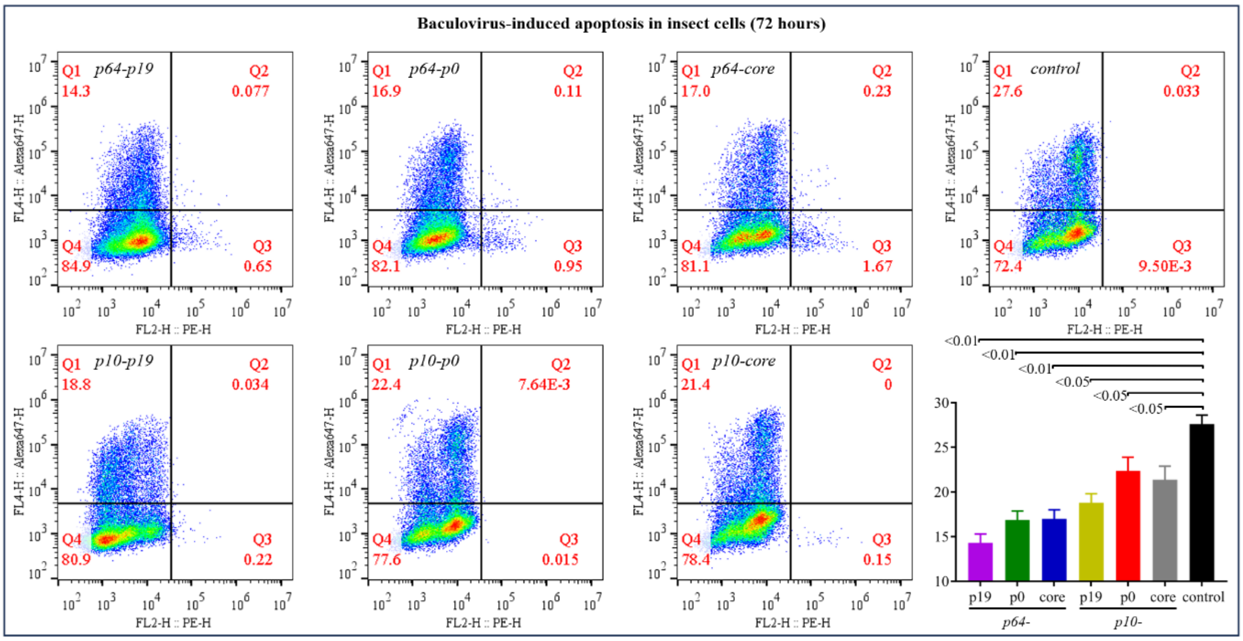

Baculovirus-induced apoptosis in insect cells (72 hours)
p64-p19
Q1 14.3
Q2 0.077
Q4 84.9
Q3 0.65
p64-p0
Q1 16.9
Q2 0.11
Q4 82.1
Q3 0.95
p64-core
Q1 17.0
Q2 0.23
Q4 81.1
Q3 1.67
control
Q1 27.6
Q2 0.033
Q4 72.4
Q3 9.50E-3
p10-p19
Q1 18.8
Q2 0.034
Q4 80.9
Q3 0.22
p10-p0
Q1 22.4
Q2 7.64E-3
Q4 77.6
Q3 0.015
p10-core
Q1 21.4
Q2 0
Q4 78.4
Q3 0.15
FL4-H :: Alexa647-H
FL2-H :: PE-H
<0.01
<0.01
<0.01
<0.05
<0.05
<0.05
p19
p0
core
p19
p0
core
control
p64-
p10-

D

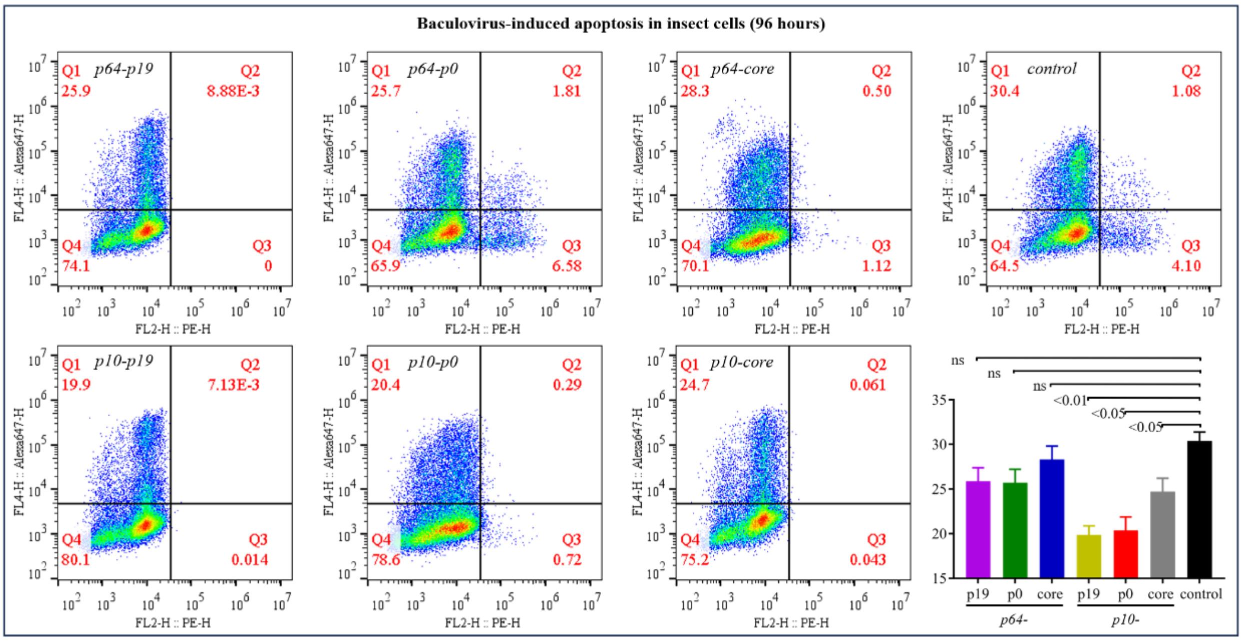

Baculovirus-induced apoptosis in insect cells (96 hours)
p64-p19
Q1 25.9
Q2 8.88E-3
Q4 74.1
Q3 0
p64-p0
Q1 25.7
Q2 1.81
Q4 65.9
Q3 6.58
p64-core
Q1 28.3
Q2 0.50
Q4 70.1
Q3 1.12
control
Q1 30.4
Q2 1.08
Q4 64.5
Q3 4.10
p10-p19
Q1 19.9
Q2 7.13E-3
Q4 80.1
Q3 0.014
p10-p0
Q1 20.4
Q2 0.29
Q4 78.6
Q3 0.72
p10-core
Q1 24.7
Q2 0.061
Q4 75.2
Q3 0.043
FL4-H :: Alexa647-H
FL2-H :: PE-H
ns
ns
ns
<0.01
<0.05
<0.05
p19
p0
core
p19
p0
core
control
p64-
p10-

E

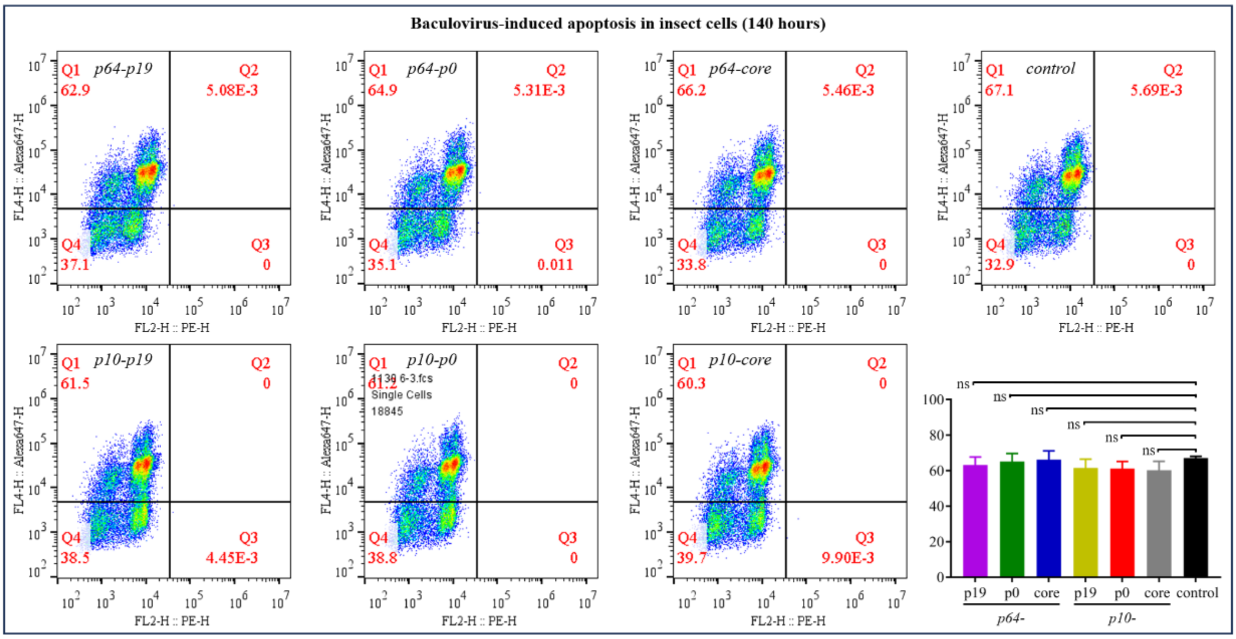

Baculovirus-induced apoptosis in insect cells (140 hours)
Q1 p64-p19 62.9
Q2 5.08E-3
Q4 37.1
Q3 0
Q1 p64-p0 64.9
Q2 5.31E-3
Q4 35.1
Q3 0.011
Q1 p64-core 66.2
Q2 5.46E-3
Q4 33.8
Q3 0
Q1 control 67.1
Q2 5.69E-3
Q4 32.9
Q3 0
Q1 p10-p19 61.5
Q2 0
Q4 38.5
Q3 4.45E-3
Q1 p10-p0 61.2
1130 6-3.fcs
Single Cells
18845
Q2 0
Q4 38.8
Q3 0
Q1 p10-core 60.3
Q2 0
Q4 39.7
Q3 9.90E-3
FL4-H :: Alexa647-H
FL2-H :: PE-H
ns
p19 p0 core p19 p0 core control
p64-
p10-
